# Clinical and Molecular Findings in Patients with Knobloch Syndrome 1: Case Series Report

**DOI:** 10.3390/genes15101295

**Published:** 2024-10-01

**Authors:** Tatyana Vasilyeva, Vitaly Kadyshev, Olga Khalanskaya, Svetlana Kuznetsova, Sofya Ionova, Andrey Marakhonov, Rena Zinchenko

**Affiliations:** Research Centre for Medical Genetics, 115522 Moscow, Russia; mgnc@med-gen.ru (V.K.); svetakuz123321@gmail.com (S.K.); sofya.aydarovna.g@med-gen.ru (S.I.); marakhonov@generesearch.ru (A.M.); renazinchenko@mail.ru (R.Z.)

**Keywords:** *COL18A1*, Knobloch syndrome, myopia, vitreoretinal atrophy, occipital encephalocele, connective tissue dysplasia

## Abstract

Background/Objectives: Knobloch syndrome 1 (KS) is an autosomal recessive inherited ocular syndrome characterized by a combination of high myopia, vitreoretinal degeneration, and occipital encephalocele. KS is caused by biallelic pathogenic variants in the *COL18A1* gene. Diagnosing KS can be challenging due to its clinical heterogeneity and the rarity of the syndrome. Methods: We conducted comprehensive clinical and instrumental ophthalmological examinations, whole-exome sequencing, Sanger sequencing, and segregation analysis to evaluate affected families. Results: Two patients presenting with high myopia, low visual acuity, chorioretinal atrophy, and occipital skin/skull defects were diagnosed with Knobloch syndrome 1 (KS). In Case 1, a 14-year-old boy, the *COL18A1* variants identified were c.2673dup and c.3523_3524del in a compound heterozygous state. Case 2 involved a 3-year-old girl, the c.1637_1638dup and c.3523_3524del variants were identified in a compound heterozygous state. In Case 3, a retrospectively observed boy of 3 y.o. with KS, the variants c.929-2A>G and c.3523_3524del were defined earlier. Conclusions: We confirmed KS molecularly in two novel families. Additionally, in Case 3 of a retrospectively analyzed third family and in both novel cases, one of the biallelic causative variants was the same known 2bp deletion in exon 40 of the collagen XVIII gene. Cases 1 and 3 were characterized by connective tissue dysplasia features and a pathognomonic Knobloch triad. No neurological manifestations and no trends in the genotype–phenotype relationship were found. The heterogeneity of phenotype in the case series is likely to be the result of further factors and/or genetic background.

## 1. Introduction

Even though next-generation sequencing has become a routine practice in medical genetics, we continue to appreciate its capacity to identify rare hereditary pathologies and provide solutions for complex diagnostic challenges.

Knobloch syndrome 1 (KS, OMIM #267750) is one of the rarest inherited syndromes. According to Orphanet data, it occurs in less than one case per one million people (https://www.orpha.net/ accessed on 1 January 2024). KS is defined by a characteristic triad of pathognomonic features: high myopia, vitreoretinal degeneration often accompanied by retinal detachment, and occipital encephalocele [1]. KS is inherited in an autosomal recessive manner and results from biallelic pathogenic variants in the *COL18A1* gene. The protein encoded by *COL18A1*, collagen XVIII, is a basement membrane proteoglycan expressed in the epithelium and endothelium of various tissues, particularly in the blood vessels and cartilage [2,3]. Collagen XVIII plays an important role in the embryonic development of retinal vessels, ocular formation, neuronal migration, and neural tube closure [4].

Differential diagnosis of KS is challenging due to the variability of its clinical presentation. Occipital abnormalities can manifest as either encephalocele, a bone defect, or only skin aplasia [5]. An ophthalmic phenotype may include additional anterior and/or posterior segment anomalies such as lens subluxation, cataracts, iris atrophy, iris synechiae, macular “punched” lesions, pigment redistribution in the fundus, albinotic fundus, and other ocular signs [6]. Furthermore, high myopia associated with connective tissue abnormalities can also be indicative of other rare inherited collagenopathies, e.g., Marfan syndrome or Stickler syndrome [7].

In this study, we present the genetic testing results and clinical characteristics of two newly identified KS patients, along with a previously diagnosed patient in our center. Notably, all three KS cases share the same *COL18A1* variant, which appears to be a recurrent mutation associated with this condition.

## 2. Materials and Methods

### 2.1. Patient Cohort and Samples

Three patients with Knobloch syndrome 1 (KS1) were recruited during genetic counseling at the Research Centre for Medical Genetics (Moscow, Russia). The affected probands and their healthy relatives underwent a comprehensive ophthalmic examination. These assessments included autorefractometry, Snellen chart estimation of best corrected visual acuity (BCVA), slit lamp biomicroscopy, photography, tonometry, ocular ultrasonography, optical coherence tomography (OCT), fundus ophthalmoscopy, and electroretinography.

Written informed consent was obtained from all participants (or their legal guardians) prior to their inclusion in the study. The study was conducted in accordance with the Declaration of Helsinki and approved by the Institutional Review Board of the Research Centre for Medical Genetics, Moscow, Russia (protocol no. 2017-4/1, dated 4 May 2017).

### 2.2. Clinical Genetic Testing

#### 2.2.1. DNA Purification

Genomic DNA was isolated from peripheral blood leukocytes using the Wizard^®^ Genomic DNA Purification Kit (Promega, Madison, WI, USA) according to the manufacturer’s recommended method.

#### 2.2.2. High-Throughput Sequencing

To identify the causative variant in one of the index patients, whole exome sequencing (WES) was recommended. WES was conducted using a BGISEQ-500 instrument, achieving an average on-target coverage of 146×, with library preparation performed using the MGIEasy Exome Capture V4 kit (BGI, Shenzhen, China) (Genomed Ltd., Moscow, Russia). Data analysis was performed using an in-house software pipeline as previously described [8]. This included quality control of raw reads using the FastQC tool (v. 0.11.5) followed by read mapping to the hg19 human genome assembly (bwa mem v. 0.7.1), sorting of the alignments, and duplicate marking (Picard Toolkit v. 2.18.14). Base recalibration and variant calling were carried out using GATK3.8. Variants were annotated with the Annovar tool (v. 2018Apr16) and filtered based on functional consequences, population frequencies following the ACMG recommendations, and clinical relevance using the Human Phenotype Ontology database [9].

#### 2.2.3. Sanger Sequencing

All pathogenic *COL18A1* variants retained after WES filtering were confirmed by Sanger sequencing using the BigDye Terminator v3.1 Cycle Sequencing Kit on a 3100XL Genetic Analyzer (Thermo Fisher Scientific, Waltham, MA, USA) and specifically designed primers targeting exons 14, 32, and 40. Genetic information was referenced to human genome version hg19 and the *COL18A1* gene, refSeq: NM_001379500.1. The primers used for variant validation and segregation analysis are as follows: 5′-GTGTGCAAGCATCTTCCCGC-3′ (14F); 5′-ATTCCTGGCATTTCCCCTTCA-3′ (14R); 5′-CCCAGGCACTAGGGCATTTC-3′ (32F); 5′-CTGGGATGTCCAGAGTTGCT-3′ (32R); 5′-CTCTGACCTGGCAGCGTATG-3′ (40F); 5′-ACTGGACTGACCCACCTTGA-3′ (40R). Parental studies were conducted to confirm that the identified variants were inherited on different alleles (in trans) from each parent. 

## 3. Results

KS was confirmed in two families through medical examination and genetic testing, while data from a third family, previously diagnosed, are included here as part of a case series of KS patients identified at the RCMG (Research Centre for Medical Genetics) [10]. All patients exhibit typical ocular abnormalities and have normal postnatal neuromotor development. A summary of the key clinical features is provided in Table 1. 

Biallelic variants in the *COL18A1* gene were identified in all three families, as shown in Table 1. Whole exome sequencing revealed no additional potentially pathogenic variants in *COL18A1* or in any other genes associated with retinal dystrophies and/or connective tissue disorders in these families.

### 3.1. Case 1. Family K

Patient K, a 14-year-old boy, has been diagnosed with high myopia, strabismus, ectopia lentis, nystagmus, vitreoretinal dystrophy, and severe visual impairment, resulting in his classification as visually disabled. He currently reports difficulty with both distance and near vision in both eyes, as well as decreased contrast sensitivity. 

#### 3.1.1. Medical History Data

The mother gave birth to her son at the age of 29, and the father was the same age at the time of delivery. Both parents are healthy urban residents from the Far East Federal region, Caucasian, of Russian descent, and do not belong to any ethnic minority. The patient was born via an unremarkable delivery during the first pregnancy and first delivery, at term, weighing 3600 g and measuring 56 cm in length. He achieved developmental milestones on schedule. 

**Figure 1:**
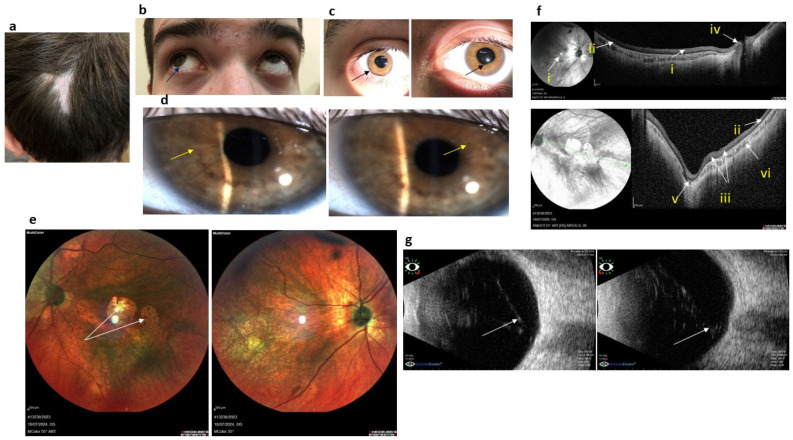
Results of examination of a boy with KS (Case 1, m 14 y.o., Family K). (**a**) Photo of the occipital part. The white arrow points to scarring hair loss that arose after encephalocele surgery. (**b**) Photo of the patient’s eyes. Arrows depict deviation of the eyeball. (**c**) Arrows depict iridocorneal synechia and pupil shape anomalies. (**d**) Slit lamp photo. Arrows depict the iris without crypts and cornea pigment precipitates. (**e**) Ophthalmoscopy results. Arrows point to chorioretinal dystrophic foci in the central part of the patient’s left eye. (**f**) OCT results. Arrows depict right foveal umbo absence (i), thinned retina (ii), undifferentiated inner retinal layers (iii), thin nerve fiber layer (iv), destructed retinal outer layer (v), and thinned choriocapillaris layer (vi). (**g**) Ultrasonic eye examination results. Arrows depict bilateral fiber cords in the central parts of the retina.

Medical history indicates that the patient has experienced nystagmus, which diminished with age, as well as myopia, strabismus, and optic nerve atrophy from birth. At 4 months old, he underwent surgery for encephalocele; at 3 years old, he had surgery to correct strabismus; at 5 years old, he received an injection of a vasculogenesis excitor into both eyeballs to restore optic nerve vasculature. At 14 years old, he received a subtenon injection of retard steroid. In the occipital region, the patient has post-surgical scar-related hair loss (Figure 1a).

#### 3.1.2. Somatic and Neurologic Status

The child exhibits no mental or physical abnormalities and is socially well adjusted. He has an asthenic body constitution, standing 181 cm tall and weighing 64 kg. Notably, he presents with funnel chest deformity and moderate facial dysmorphia, characterized by an elongated face, low forehead, downward slant of the palpebral fissures, a prominent nasal bridge, and a pronounced chin. The referring diagnosis of the patient is connective tissue dysplasia.

#### 3.1.3. Ophthalmological Status

The patient’s best-corrected vision acuity (BCVA) is 0.04 in the right eye and 0.05 in the left eye. Autorefractometry indicates high myopia with complex myopic astigmatism (OD sph—11.25 cyl—2.75 ax 1 OS sph—10.75 cyl—10.25 ax 159) and an outward deviation of the eyeballs by 10 degrees (Figure 1b). He exhibits horizontal small-swinging nystagmus. Tonometry shows normal intraocular pressure (OD 20 mm Hg, OS 19 mm Hg). 

Slit lamp examination reveals significant anterior segment abnormalities, including diffuse pigment precipitates on the posterior surface of the corneal endothelium, subathrophic iris without crypts, iridocorneal synechiae, weakened pupillary light reflex, cortical lens opacity in the paraoptical zone, and a destructed and opalescent vitreous body (Figure 1c,d). Fundus examination shows pale pink, uniformly colored optic discs with blurred edges covered by glial tissue, as well as enlarged retinal veins. The macular reflex is diminished, and two atrophic foci are observed at the level of choriocapillaris and pigment epithelium in the center of the left eye, each approximately one and a half times the diameter of the optic nerve disc (Figure 1d). Additionally, pigmented atrophic foci are present in the retinal periphery, and vitreoretinal cords are detected in the lower periphery quadrants (Figure 1e). 

OCT findings are presented in Figure 1f. The OCT reveals the absence of the right foveal umbo, chorioretinal atrophy in the macular region, visible sclera, drastically thinned retina, increased retinal reflexivity, indistinct inner retinal layers, a thin nerve fiber layer, disrupted outer retinal layer, and severely thinned choriocapillaris layer. 

Ultrasonic eye examination indicates an enlarged anteroposterior eye axis (OS–26.5 mm, OD—27.0 mm). B-scan imaging shows severe vitreous body destruction and bilateral fiber cords in the central retina (Figure 1g). Previous electroretinography (ERG) demonstrated bilateral dysfunction of both retinal rods and cones, with decreased subnormal ERG responses. The patient does not experience visual impairment at dusk, does not exhibit photophobia, and has abnormal trichromasia for color perception. Dark adaptation in his eyes is not significantly impaired. 

#### 3.1.4. Genetic Testing

**Figure 2:**
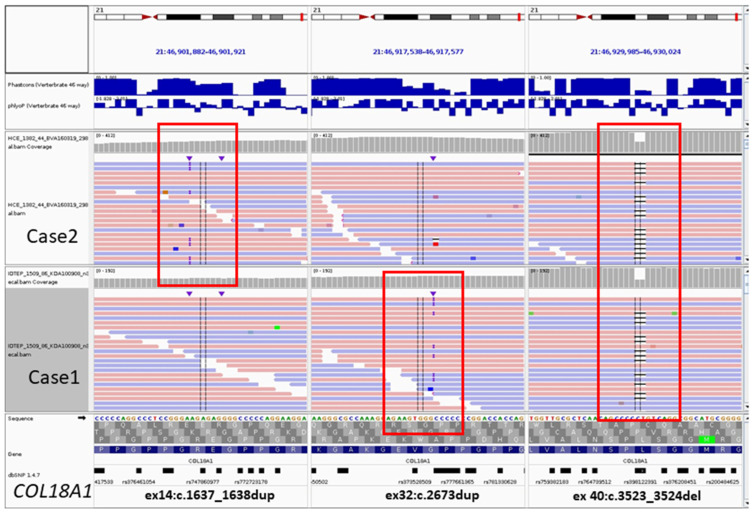
Whole exome sequencing results for Cases 1 and 2. Red squares frame the patients’ variants, which are shown at the bottom. Detected genotypes according to NM_001379500.1(*COL18A1*) are c.[2673dup];[3523_3524del] in Case 1 and c.[1637_1638dup];[3523_3524del] in Case 2.

Whole exome sequencing identified two known heterozygous variants in *COL18A1*: NM_001379500.1(*COL18A1*):c.2673dup p.(Gly892Argfs*9) (HGMD:CI092465) [11] and c.3523_3524del p.(Leu1175Valfs*72) (HGMD:CD023246) [12] (Figure 2). The patient’s clinical and genotype data are summarized in Table 2.

**Figure 3:**
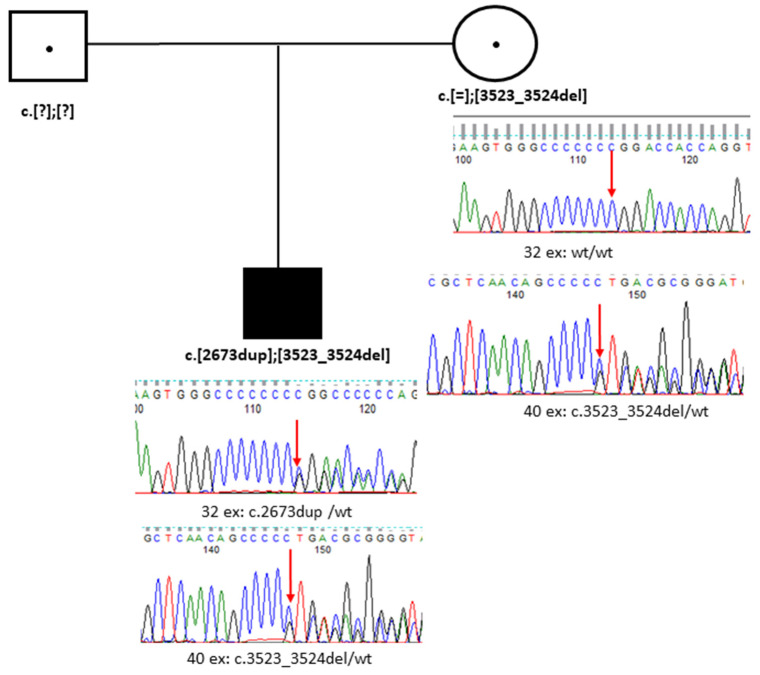
Segregation analysis by Sanger sequencing in Family K (Case 1). Note: A mother has only a variant of proband in the heterozygous state.

Sanger sequencing confirmed that the patient’s unaffected mother is heterozygous for one of these variants, specifically the common deletion c.3523_3524del in exon 40 (Figure 3). A father was unavailable for genetic testing.

The c.2673dup variant is located in exon 32 out of 42 and results in a frameshift, creating a premature stop codon, p.(Gly892Argfs*9). According to the NMDescpredictor tool [13], this variant may trigger nonsense-mediated decay (NMD) to eliminate the aberrant RNA, suggesting it is a likely loss-of-function (LOF) variant. Similarly, the c.3523_3524del variant in exon 40 out of 42 also causes a frameshift and premature stop codon formation p.(Leu1175Valfs*72). This variant is also predicted to undergo NMD, indicating a potential LOF effect, according to the NMDescpredictor tool [13]. 

### 3.2. Case 2. Family B

#### 3.2.1. Medical History Data

Patient B is a 3-year-old girl diagnosed with high myopia, choroid atrophy, and an albinotic fundus. She currently reports poor distance vision and rapid myopia progression in the right eye, with an increase in three diopters over the past year. Her vision decline began gradually and was first noted at 1.8 years of age.

Both parents are healthy. The mother gave birth to her daughter at 31 years old during her first pregnancy. The father was the same age at the time of delivery. The parents are urban residents of Russia (Central and East Siberian regions), Caucasian, of Russian descent, and do not belong to any ethnic minority. The patient was born at term from an unremarkable first pregnancy and delivery, weighing 2940 g and measuring 53 cm in length. She reached developmental milestones on time.

The patient has a normosthenic body constitution and does not present with any somatic abnormalities. 

#### 3.2.2. Ophthalmological Status

The patient’s BCVA is 0.1 in both eyes. Examination of the anterior eye segment reveals normal conjunctiva, cornea, iris, lens, pupil, and anterior chamber in both eyes, as well as a normal vitreous body. Fundus examination shows a pale pink optic disc with clear boundaries and a centrally located vascular bundle. The vascular course is regular. The macular reflex is weak and intermittent, and the peripheral retina is transparent, with visible choroidal vessels and dissociated pigment. The patient’s referring diagnosis is high myopia and ocular albinism.

#### 3.2.3. Genetic Testing

Whole exome sequencing identified one known variant, (NM_001379500.1(*COL18A1*):c.3523_3524del p.(Leu1175Valfs*72), HGMD:CD023246) [12] and one novel variant, c.1637_1638dup p.(Gly547Argfs*178), for which the patient is a compound heterozygote. The WES results are illustrated in Figure 2. The patient’s clinical features and her genotype are summarized in Table 2 (Patient/Family B).

**Figure 4:**
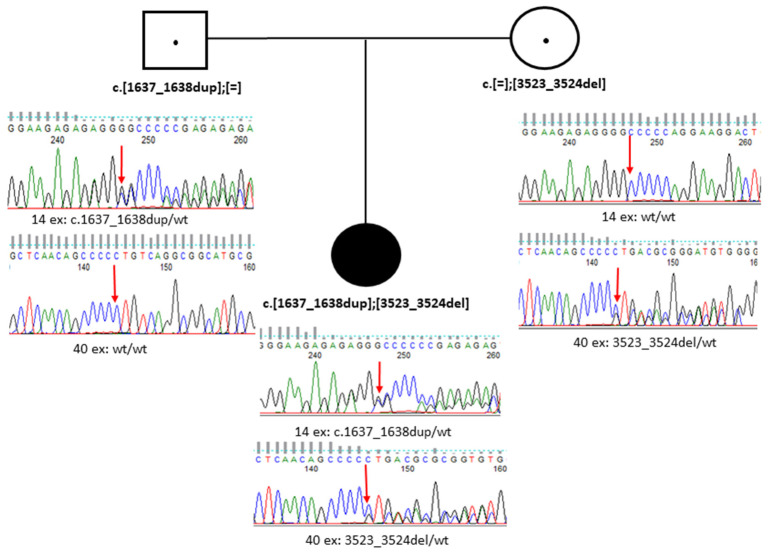
Sanger sequencing results in Family B (Case 2). Note: A father and a mother are each shown to be a heterozygous carrier of one of their daughter’s pathogenic variants.

Sanger sequencing confirmed that the novel c.1637_1638dup variant is present in a heterozygous state in the patient’s healthy mother, while the known c.3523_3524del variant is present in a heterozygous state in the patient’s healthy father (Figure 4).

The c.1637_1638dup variant is located in exon 14 out of 42 and causes a frameshift leading to the formation of a premature stop codon, p.(Gly547Argfs*178). This variant is likely to undergo NMD, making it a probable LOF variant [13]. The variant has not been identified in healthy individuals according to gnomAD v.4.1.0 database, and it should be classified as a likely pathogenic variant based on ACMG criteria PM2 and PVS1 [14]. 

The *c.3523_3524del* variant, located in exon 40 out of 42, also results in a frameshift and premature stop codon formation, p.(Leu1175Valfs*72). Similar to the previous case, this variant is predicted to undergo NMD and is likely an LOF variant. 

### 3.3. Case 3. Family OB

#### 3.3.1. Medical History Data

We also revisited a previously reported case of KS diagnosed at our center in 2018 [10]. There is no demographic information available on the family. The affected child was born from the third pregnancy; the first pregnancy was medically terminated, and the second pregnancy ended in miscarriage. 

Patient OB, a 3-year-old boy, presented with high myopia, low visual acuity, choroid and optic nerve atrophy, nystagmus, and dissociated pigment in the eye fundus. Additional clinical features included an asthenic body constitution, funnel chest deformity, thin skin, joint hypermobility, blue sclera, midface hypoplasia, and a small posterior cranial fossa cyst. 

#### 3.3.2. Genetic Testing

Whole exome sequencing identified two known heterozygous variants NM_001379500.1(*COL18A1*): c.929-2A>G p.(?), (HGMD:CS092463) [11] and c.3523_3524del (HGMD: CD023246). The patient’s clinical description and genotype information are included in Table 2 (Case 3, Family OB).

**Figure 5:**
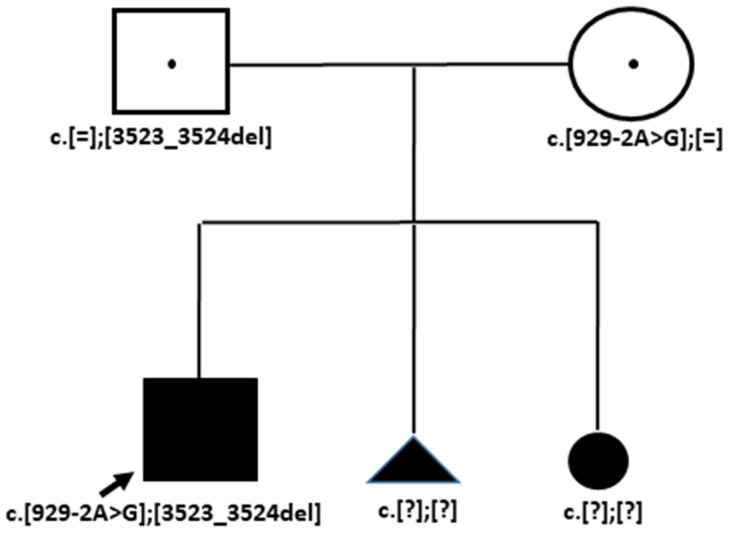
Segregation analysis results in Family OB (Case 3). Note: A father and a mother are each shown to be a heterozygous carrier of one of their son’s pathogenic variants.

Sanger sequencing confirmed that the c.929-2A>G variant is present in the heterozygous state in the patient’s healthy mother, while the c.3523_3524del variant is present in the heterozygous state in the patient’s healthy father (Figure 5).

The c.929-2A>G variant is located in intron 5 and has the potential to disrupt normal splicing, which may lead to a shortening of exon 6 by five base pairs. This alteration could result in a frameshift and the formation of a premature termination codon (PTC). Consequently, it is likely that NMD will eliminate the aberrant RNA, indicating that this variant is an LOF variant [15]. 

The c.3523_3524del variant located in exon 40 out of 42 is the same as those identified in previous cases (as noted above). 

## 4. Discussion

Three unrelated patients—a 14-year-old boy, a 3-year-old girl, and a 3-year-old boy—were referred to our center (RCMG) with a diagnosis of coincidental high myopia, low vision, vitreoretinal destruction (without retinal detachment at the moment of diagnosis), and an occipital skull defect The clinical presentations were heterogeneous, and the initial diagnoses varied among the patients, differing from the eventual diagnosis of Knobloch syndrome (KS) established through WES results. All three patients were found to have biallelic LOF variants in the *COL18A1* gene.

The *COL18A1* gene encodes an important component of basement membranes and is a regulator of eye embryogenesis. The cleavage of the C-terminal portion of COL18A1 produces endostatin, which plays a role as an angiogenesis inhibitor. Additionally, the N-terminal region of COL18A1 contains a frizzled-like domain that exhibits WNT-inhibiting activity and thrombospondin motif [16]. 

Ocular affections in KS patients are believed to result from anomalies in retinal vascularization that occur during embryogenesis. A deficiency in COL18A1 is likely to cause delayed regression of hyaloid vessels and result in conditional hyperoxia, whereas normal embryonic retinal vascularization is driven by hypoxia-induced expression of vascular endothelial growth factor (VEGF) by astrocytes [17]. Furthermore, it is suggested that vitreous collagen fibers along the inner limiting membrane are reduced in number in KS patients, which may adversely affect the prognosis for retinal detachment [12]. 

The vast majority of pathogenic variants in the *COL18A1* gene are frameshift mutations, accounting for 28 out of 43 damaging variants documented in the HGMD database [18]. It can be inferred that all four identified variants in our study lead to loss of function, resulting in null *COL18A1* expression due to the presence of biallelic pathogenic variants in all three patients.

To date, no functional analyses have been conducted for any of the identified *COL18A1* variants. The predominant molecular pathogenic mechanism associated with Knobloch Syndrome 1 (KS) is loss-of-function mutations in the *COL18A1* gene. Forty-four out of the fifty-seven known variants listed in the HGMD database (v.2022.1) are classified as LOF. Notably, LOF *COL18A1* variants have never been reported in the homozygous state in samples from healthy individuals (https://gnomad.broadinstitute.org/gene/ENSG00000182871?dataset=gnomad_r4m, accessed on 1 January 2024). Furthermore, immunohistochemical analyses of skin biopsies from KS patients with null mutations have demonstrated the absence of type XVIII collagen expression [11]. The impact of the loss of *CO18A1* expression on downstream-regulated genes remains to be elucidated. Recently, an induced pluripotent stem cell (iPSC) line derived from KS patients has been generated, which could facilitate investigations into the pathomolecular mechanisms of the syndrome and potential treatment strategies in vitro [19].

The diverse clinical manifestations of KS have been extensively discussed in the literature [6,20]. In one of our patients, a 3-year-old girl (Case 2/Family B), examination revealed a transparent retina with visible choroidal vessels, leading to an initial diagnosis of ocular albinism. Albinotic features in the eye fundus have been previously reported [5,6] and may be attributable to the thin retina, a condition associated with *COL18A1* defect [16]. Notably, this girl does not exhibit severe vitreoretinal degeneration, nystagmus, or retinal detachment, and her degree of myopia is the least severe among our three patients. Additionally, she shows no other signs of connective tissue dysplasia aside from a small occipital dermoid cyst (Table 1, Figure 1). In contrast, Case 3 from Family OB displays very high myopia (18 diopters), significantly low vision, choroidal, and optic nerve atrophy. Meanwhile, Case 1 from Family K presents with severe vitreoretinal dystrophy, optic nerve atrophy, and very low vision from birth, alongside notable anterior segment abnormalities such as a cryptless iris with synechiae and cataracts. Such anterior segment abnormalities are indeed quite common in KS patients [6]. 

The two patients (Cases 3 and 1) exhibit not only eye afflictions but also notable features of connective tissue disorder. Specifically, the boy from Case OB presents with blue sclera, an asthenic body constitution, funnel chest, joint hypermobility, thin skin, midface hypoplasia, and an occipital fossil cyst. Similarly, the boy from Case K demonstrates an asthenic constitution with thin skin, pectus excavatum, joint hypermobility, facial deformities, and an occipital encephalocele. 

The signs of connective tissue dysplasia in Case 1 initially suggested a diagnosis of Marfan syndrome. Marfan syndrome shares several characteristics with KS, including myopia magna and ectopia lentis, often in conjunction with tall stature and an asthenic constitution. However, KS is generally not associated with a wide range of connective tissue dysplasia features, apart from occipital skull defects and joint hypermobility. Notably, previous reports have mentioned individual KS patients exhibiting additional traits, such as an atrial septal defect [21], and others presenting with a narrow face, epicanthal folds, high forehead, and temporal narrowing [22]. These observations raise the possibility of at least two explanations for the connective tissue signs noted in our KS case series. One possibility is that Cases K and OB represent instances of “double trouble”, where a second genetic variant contributing to their connective tissue features has yet to be identified. The alternative explanation is that signs of connective tissue dysplasia may indeed be more universally present in all KS patients than previously recognized. It is also plausible that both factors contribute to the phenotypic diversity observed in individuals with KS. Further genetic studies and analyses are needed to clarify these associations and enhance our understanding of KS’s clinical spectrum.

All three of our KS patients are compound heterozygous for the same known pathogenic two-base pair deletion, NM_001379500.1(*COL18A1*):c.3523_3524del. To explore possible genotype-phenotype correlations, we examined several previously reported cases of individuals who are also compound heterozygous for this variant. This particular variant is recurrent and occurs in a hotspot, as evidenced by the fact that the allele with the variant has been found on different haplotypes [12]. We excluded previously reported homozygous cases for the variant because most of those children were born from consanguineous marriages, which could complicate their phenotypes due to the influence of other genetic factors [23]. The patient’s clinical features and genotypes are summarized in Table 2.

**Table 2:** Clinical features and genotypes of cases published earlier and reported here with the same recurrent variant c.3523_3524delCT in a compound-heterozygous state with another pathogenic COL18A1 variant.

Features/Patients	Patient 1 [24]	KS4.1 [12]	KS4.2 [12]	KS5 [12]	Case K (This Study)	Case B (This Study)	Case OB [10]
*COL18A1* genotypes	c.2960_2969 dup	c.1238insA	c.1238insA	c.2105delC	c.2673dup	c.1637_1638 dup	c.929–2A>G
	c.3523_3524 delCT	c.3523_3524 delCT	c.3523_3524 delCT	c.3523_3524 delCT	c.3523_3524 delCT	c.3523_3524 delCT	c.3523_3524 delCT
SEX	F	F	M	M	M	F	M
AGE	2 y.o.	21 y.o.	13 y.o.	6 y.o.	14 y.o.	3 y.o.	3 y.o.
Myopia magna	Yes	Yes	Yes	Yes	Yes	Yes	Yes
Visual acuity (with optical correction)	0.9 logmar and less than 1.3 logmar Decimal 0.125 и 0.005	Blind (at age 5)	20/200 best corrected (both eyes)	Blind (at age 1 year)	Low visual acuity	Low visual acuity	Low visual acuity
Nistagmus	Yes	n/m ^1^	n/m	n/m	Yes	No	Yes
Eye fundus, retinal defect	Irregular macular region in the right fundus and atrophic lesion in the left fundus, thin retina with visible choroidal vessels	Retinal detachment, vitreoretinal degeneration	Retinal detachment, vitreoretinal degeneration	Retinal detachment	Tessellated fundus, thin retina with visible choroidal vessels, atrophic macula and optic nerve	Tessellated fundus, thin retina with visible choroidal vessels	Atrophic choroidea and optic nerve, tessellated fundus
Macula functionality	ERG showed the presence of scotopic and photopic responses, but severely reduced amplitudes and delayed latencies	n/m	n/m	n/m	Weakened reflex from the macula, retinal rod and cone dysfunction, trichromasia, and decreased subnormal ERG	Weakened reflex from the macula	n/m
Anterior segment	No	n/m	n/m	n/m	Atrophic iris, ectopia lentis, cataract	No	No
Occipital defect	Meningoencephalocele	Encephalocele	Encephalocele	Encephalocele	Cranial hernia	Dermoid cyst	Small posterior cranial fossa cyst
Additional connective tissue displasia features	n/m	No	No	No	Asthenic body constitution, funnel chest deformity, thin skin, joint hypermobility, blue sclera, midface hypoplasia	No	Asthenic body constitution, funnel chest deformity, thin skin, joint hypermobility, blue sclera, midface hypoplasia

Note: ^1^ n/m—not mentioned/unknown.

All seven patients are compound heterozygous for two LOF *COL18A1* variants. They exhibit varying disease manifestations and severities. Notably, none of the three patients from our study experienced retinal detachment, although two of them displayed additional features of connective tissue dysplasia. Overall, the disease course appears to be severe across all seven patients; two experienced early blindness, and three had retinal detachment. Among the patients, Case B appears to exhibit the least severe manifestations. Her second variant c.1637_1638dup, is also an LOF variant, consistent with the other variants identified in this cohort. 

Thus, we observe diverse disease manifestations and severity among patients who are compound heterozygous for null alleles, including the LOF variant c.3523_3524delCT. It is suggested that *COL18A1* null alleles lead to severe ocular involvement [16]. The unique clinical features observed in these cases likely relate to unknown genetic background and/or modifying factors. The severe progression of the disease course in *COL18A1* homozygous patients born from consanguineous marriages further supports the influence of genetic background on the severity of the KS phenotype [25]. 

For instance, epilepsy was reported in only one out of three siblings who were homozygous for the same 2 bp deletion and born from a consanguineous marriage [26]. In another family, one out of two siblings homozygous for the c.3523_3524delCT variant had a medical history that included Walker malformation, occipital meningocele, polymicrogyria, intractable epilepsy, and severe behavioral changes. In contrast, the other sibling was neurologically healthy but presented with significant ocular issues, including high myopia and macular coloboma, along with severely impaired cone and rod responses [5]. Additionally, a case of “double trouble” was reported, highlighting the potential influence of other genetic factors on phenotype severity, where a patient had co-occurring pathogenic variants in the *COL9A2* and *COL18A1* genes [23].

Currently, there is no universally effective treatment for KS patients. As with other inherited congenital developmental disorders, a comprehensive treatment strategy is essential. This may encompass gene therapy approaches alongside palliative care. Gene therapy could focus on restoring impaired biological functions, addressing issues such as neonatal vascular deficits, pathogenic neovascularization, and wet age-related macular degeneration often observed in KS patients. Additionally, enhancing the stability and maintenance of the retina vessel could be a vital goal of these therapeutic interventions. 

Potential treatment options for KS could include various natural agents and gene therapy approaches. For instance, a derivative of the plant alkaloid annomontine has shown promise in inhibiting endothelial inflammation and abnormal angiogenesis by targeting CDC2-like kinases and WNT/β-catenin signaling [27]. Additionally, retinoid medications may help reduce choroidal sprouting [28]. 

Gene therapy strategies could involve the use of microRNA-376b-3p, which is thought to regulate glutamine metabolism in endothelial cells and suppress choroid neovascularization [29]. The pro-angiogenic retinal environment, characterized by the lack of endostatin inhibition and associated dysregulation of proteostasis and mitochondrial dysfunction, represents a potential target for treatment [30,31]. However, direct endostatin treatment is inappropriate, as its overexpression is also pathogenic. Instead, restoring full-length COl18A1 function may be more effective given its various biological roles [32]. Furthermore, anti-VEGF therapy is considered inadequate due to incomplete response observed in some patients [33]. Gene therapy approaches include the development of in vitro choroid models for drug screening and vector testing [34,35,36]. Continued research is necessary to better define gene therapy targets for treating KS patients.

## 5. Conclusions

We described three families with Knobloch Syndrome 1 who share the same recurrent variant in the *COL18A1* gene. Despite this genetic similarity, their clinical presentations vary widely, although they are all characterized by severe visual impairment. Notably, two patients in this study exhibited distinct signs of connective tissue dysplasia, suggesting that additional, yet unidentified, genetic factors may contribute to their atypical KS phenotypes. In an effort to identify commonalities or differences among patients with the same 2-bp deletion in exon 40 of the *COL18A1* gene, we reviewed the literature and identified four more KS patients who are compound heterozygous for this recurrent variant. However, we did not observe any expected trends in genotype-phenotype correlations. One possible explanation for this lack of correlation is that all identified pathogenic *COL18A1* variants result in loss of function. Consequently, the severity of the phenotype in KS patients appears to be more influenced by individual genetic backgrounds rather than solely by the specific *COL18A1* mutations.

## Figures and Tables

**Table 1:** Clinical and molecular genetic findings in three KS patients.

Family with a Patient	Case 1/Family K	Case 2/Family B	Case 3/Family OB
Sex	M	F	M
Age	14 y.o.	3 y.o.	3 y.o.
Retinal detechment	No	No	No
Myopia (diopter—D)	>10–13 D, compound myopic astigmatism	>6 D, compound myopic astigmatism	18 D, compound myopic astigmatism
Strabismus	Nonconcomitant strabismus	No	n/m
Lens subluxation	No	No	n/m
Cataract	Yes	No	No
Vitreoretinal destruction	Yes, central eyeball fibers	n/m ^1^	n/m
Visual acuty	0.05	0.1	0.09
Choroid atrophy	Yes	Yes	Yes
Optic nerve atrophy	Optic nerve partial atrophy	Pale-pink with clear boundaries	Decolorized optic disk
Macula reflex abnormality	Weak reflex	Weak/intermittent reflex	n/m
Nistagmus	Yes	No	Yes
Fundus pigment dissociation	Yes	Yes	Yes
Occipital skull defect	Osseous tissue defect 3.3 mm, meningocele 1.6 × 0.95 mm	Dermoid cyst, occipital hemangioma 0.5 cm	Small posterior cranial fossa cyst
Neurological phenotype	No	No	No
Psychomotor development	No	No	No
Asthenic constitution	Yes	n/m	Yes
Pectus excavatum (funnel chest)	Yes	Yes	Yes
Thin skin	n/m	n/m	Yes
Joint hypermobility	Yes	n/m	Yes
Blue eye sclera	n/m	n/m	Yes
Face deformity	Elongated face, low forehead, downward palpebral slant, massive chin, large nasal bridge	n/m	Hypoplastic midface
Genotype NM_001379500.1(*COL18A1*)	c.[2673dup]; [3523_3524del]	c.[3523_3524del]; [1637_1638dup]	c.[3523_3524del]; [929-2A>G]

Note: ^1^ n/m—not mentioned/unknown. Case 3/Family OB was described earlier [10].

## Data Availability

The datasets used and/or analyzed during the current study are available from the corresponding author upon reasonable request.
